# Hardwiring of fine synaptic layers in the zebrafish visual pathway

**DOI:** 10.1186/1749-8104-3-36

**Published:** 2008-12-16

**Authors:** Linda M Nevin, Michael R Taylor, Herwig Baier

**Affiliations:** 1 Department of Physiology, University of California, San Francisco, 1550 4th Street, San Francisco, CA 94158, USA; 2 Current address : Chemical Biology and Therapeutics, St Jude Children's Research Hospital, 262 Danny Thomas Place, MS-1000, Memphis, TN 38105, USA

## Abstract

**Background:**

Neuronal connections are often arranged in layers, which are divided into sublaminae harboring synapses with similar response properties. It is still debated how fine-grained synaptic layering is established during development. Here we investigated two stratified areas of the zebrafish visual pathway, the inner plexiform layer (IPL) of the retina and the neuropil of the optic tectum, and determined if activity is required for their organization.

**Results:**

The IPL of 5-day-old zebrafish larvae is composed of at least nine sublaminae, comprising the connections between different types of amacrine, bipolar, and ganglion cells (ACs, BCs, GCs). These sublaminae were distinguished by their expression of cell type-specific transgenic fluorescent reporters and immunohistochemical markers, including protein kinase Cβ (PKC), parvalbumin (Parv), zrf3, and choline acetyltransferase (ChAT). In the tectum, four retinal input layers abut a laminated array of neurites of tectal cells, which differentially express PKC and Parv. We investigated whether these patterns were affected by experimental disruptions of retinal activity in developing fish. Neither elimination of light inputs by dark rearing, nor a D, L-amino-phosphono-butyrate-induced reduction in the retinal response to light onset (but not offset) altered IPL or tectal lamination. Moreover, thorough elimination of chemical synaptic transmission with *Botulinum *toxin B left laminar synaptic arrays intact.

**Conclusion:**

Our results call into question a role for activity-dependent mechanisms – instructive light signals, balanced *on *and *off *BC activity, Hebbian plasticity, or a permissive role for synaptic transmission – in the synaptic stratification we examined. We propose that genetically encoded cues are sufficient to target groups of neurites to synaptic layers in this vertebrate visual system.

## Background

The formation of neuronal connections is commonly thought to occur in two stages. First, genetically encoded processes, such as axon guidance by molecular cues, establish coarse connectivity by bringing presynaptic and postsynaptic partners in spatial proximity. At the tail end of this phase, cell adhesion and other cell-cell recognition mechanisms enable a large array of connections, which are thought to be, for the most part, transient and reversible. In the second stage, electrical activity is said to serve in selecting synaptic partners. According to Hebb's principle, connections between neurons with temporally correlated activity patterns are strengthened, whereas synapses between neurons with divergent activation patterns are eliminated. Correlated activity could, in principle, originate either from sensory experience or from patterns of spontaneous discharge. Activity has also been postulated to play a permissive role during the synapse consolidation by altering the cell surfaces and internal states of the synaptic partners, thereby transforming the synapse from a nascent to a mature state.

Models invoking activity as necessary for specifying connections, regardless of its exact role, make the prediction that blockade of activity should lead to abnormal or exuberant synaptic connections. Indeed, previous studies in the vertebrate central nervous system, including the mammalian visual system, have provided evidence for both instructive and permissive roles for activity in the assembly of neuronal networks [1-5]. Yet many neural circuits can form apparently correctly in the absence of activity, casting doubt on the two-step model introduced above. For instance, a mouse *munc18-1 *null mutant, which lacks synaptic transmission altogether, develops cytoarchitectonically normal neuronal connectivity throughout the brain [6]. A finer-scale study examining photoreceptor synapses in *Drosophila *mutants with aberrant neuronal signaling demonstrated that activity-independent ('hardwiring') mechanisms determine the number and position of synapses at each photoreceptor terminal [7]. In the vertebrate visual system, activity has been shown to refine the size of axonal arbors [8,9]. However, it is still unclear to what extent activity is required to organize precise laminar targeting of neurites.

In the retina, ganglion cell (GC) dendrites, amacrine cell (AC) dendrites, and bipolar cell (BC) axons converge in the inner plexiform layer (IPL), where a heterogeneous set of synapses form. These synapses are arranged in a highly regular pattern; the IPL is divided into sublaminae, or strata, each comprising a signature complement of neurites. Classic studies in the cat retina revealed that GC dendrites form an initially diffuse plexus that is refined by activity [2,10,11]. More recently, studies of the mouse retina have shown that light inputs direct GC dendrites to sublaminae [12,13]. These studies have emphasized a role for sensory experience in shaping neuronal connections in the IPL.

In contrast to the view espoused by these earlier studies, a recent time-lapse imaging study in the zebrafish retina demonstrated that the vast majority of the GC dendrites examined were confined to sublaminae from the start [14]. In addition, newly differentiated ACs project neurites directly to their target laminae, without exuberant growth or redistribution [15] and even in the absence of a major class of synaptic partners, the GCs [16]. These observations suggested that at least some zebrafish IPL neurites stratify into sublaminae from the outset of IPL development and called for an evaluation of the need for activity-based refinement in this model system.

GC axons project to 10 different visual areas in the zebrafish brain [17]. Within the largest of these targets, the optic tectum, retinofugal projections are segregated into four layers [18]. In the adult goldfish, tectal neuropil laminae are each innervated by a distinct complement of tectal dendrites [19]. In the formation of the retinotectal map, which is established within each of the four retinorecipient laminae, visual input influences the morphogenesis of GC axons, in a manner that depends on glutamatergic synaptic transmission from GCs to tectal neurons [4,8,20-22]. However, the role of retinal activity in the targeting of GC axons to tectal laminae has, to our knowledge, not been tested.

Here we show that blockage of visual experience, of correlated activity, or of chemical synaptic transmission does not prevent the emergence of apparently normal patterns of synaptic stratification in both retina and tectum of larval zebrafish.

## Results

### Cell type-specific markers demonstrate precise stratification of the IPL

**Figure 1:**
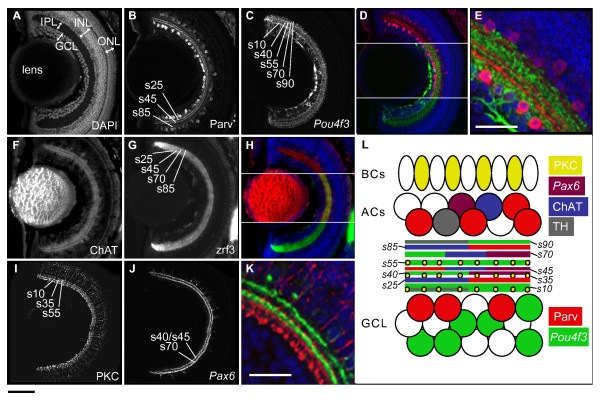
**IPL organization of the larval zebrafish retina.** Confocal images of horizontal sections of 5 dpf retina stained by immunohistochemistry or with DAPI (nuclear dye). IPL sublaminae are labeled (s10, s25, and so on). **(A) **DAPI stain shows the basic organization of the retina into GCL, IPL, INL, and ONL. **(B-E) **Neurites from Parv+ ACs (red) and *Pou4f3:mGFP+ *GCs (green) are closely apposed but reside in distinct sublaminae. **(F-H) **Neurites from ChAT+ ACs (red) overlap with zrf3 label (green) in the same sublaminae. **(I-K) **PKC+ BC axon terminals (red) and *Pax6:mGFP+ *AC neurites (green) each form three sublaminae that are closely nested but not co-localized. **(L) **Schematic of IPL organization. Cell types and their neurites are labeled according to the color code on the right. Space is shown between sublaminae for clarity. TH, tyrosine hydroxylase. Bottom left scale bar, for whole retina images, is 50 μm; bottom left scale bar in E, K is 25 μm.

At the beginning of our studies, we used known markers of retinal cell types to establish a map of the IPL sublaminae in larval zebrafish at 5 days post-fertilization (dpf; Figure 1). As in the adult zebrafish, PKC, ChAT, and Parv antisera label a population of BCs and two populations of ACs, respectively, in the inner nuclear layer (INL) and ganglion cell layer (GCL; the Parv+ cells here are displaced ACs [15]; Figure 1B, F, I). A small population of interplexiform cells in the INL are labeled with tyrosine hydroxylase (TH) antisera [23] (Additional file 1). The *Pou4f3:mGFP *transgenic reporter (previously published as *Brn3c:mGFP *[18]; mGFP is membrane targeted green fluorescent protein) labels about 40% of GCs (Figure 1C). *Pax6:mGFP *[16] labels a small population (5%) of ACs (Figure 1J). Some *Pax6:mGFP*-positive ACs co-express ChAT [16], and some co-express Parv (data not shown). The zrf3 antibody labels an unknown epitope on glial and neuronal fibers [24]. While zrf3 labels the entire IPL, the four ChAT+ sublaminae stain more strongly than the rest (Figure 1G). Zrf3 and ChAT staining were therefore used interchangeably in our subsequent studies.

Each of these labeled cell types projects neurites to two or more sublaminae of the IPL. To determine which neurites share IPL sublaminae, we performed double-labeling experiments (Figure 1D, E, H, K). In addition, we measured the positions of sublaminae relative to the IPL edges. Sublamina position can be described as a percentage of the total IPL width. By this convention, the proximal (nearest the GCL) edge of the IPL is at 0%, and the distal edge at 100%. Mumm *et al. *[14] characterized the five *Pou4f3:mGFP+ *sublaminae as s10 (the sublaminae located at 10% of the IPL width from the proximal edge), s40, s55, s70, and s90 (Figure 1C). Figure 1L is a schematic of the 5 dpf retina, including the cell types examined by us, and the nine IPL sublaminae that are their projection targets. For example, Parv+ neurons of the INL and GCL project neurites to three IPL sublaminae – two in the proximal and one in the distal half of the IPL (Figure 1A–E). Based on their position, we refer to these Parv+ bands as s25, s45, and s85 (percentages are means based on 9 measurements from 3 larvae; standard deviations ≤ 3, rounded to the nearest 5). In some sections, closely apposed bands of one type may appear fused; this is often the case for the ChAT/zrf3+ and *Pax6:mGFP+ *sublaminae and evidently depends on the sectioning angle and staining quality.

In summary, the IPL of larval zebrafish is organized quite similarly to that of other vertebrates and can be considered a miniature version of the IPL described in adult zebrafish [25,26]. Remarkably, seven of the nine sublaminae recognizable by our staining method are only 1–3 μm thick. Neurites are thus positioned with a precision that approaches the diameter of individual synaptic terminals.

### The tectal neuropil accommodates a stack of molecularly distinct layers

**Figure 2:**
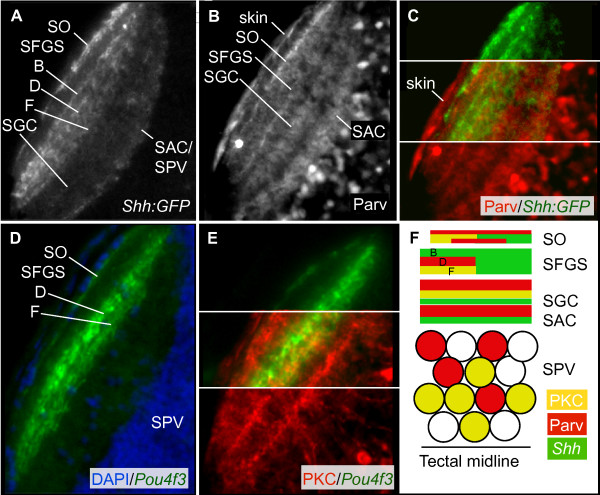
**Neuropil organization of the larval zebrafish optic tectum.** Confocal images of horizontal sections of the 5 dpf tectum stained by immunohistochemistry or with DAPI. The neuropil of one lateral half of the tectum is shown. Rostral is up. **(A) **The *Shh:GFP *transgene labels all GCs, which innervate the SO, three sublaminae of the SFGS (labeled B, D, and F), the SGC, and the SAC/SPV border. **(B) **Parv+ tectal neurites form up to five laminae, within the SO, SFGS, SGC, and SAC. The thinnest Parv+ projection is just beneath the skin, superficial to the *ShhGFP+ *SO projection, likely corresponding to the stratum marginale (SM; most visible in C). **(C) **Parv+ neurites and GC axons co-localize in the SO and SFGS, but not in the deeper tectal layers. **(D) ***Pou4f3:mGFP+ *GC axons label the SO and two sublaminae (labeled D and F) of the SFGS. **(E) **PKC+ tectal neurites are most dense in three bands in SO, SFGS, and SGC. **(F) **Schematic showing organization of the tectal neuropil. Scale bar 50 μm.

The larval zebrafish optic tectum is roughly divided into a deep cell body region and a superficial neuropil. This division is not absolute; there are also scattered cell bodies in the neuropil, most prominently a row of interneurons near the surface of the tectum. The neuropil is the arborization field for the majority of the GC axons [17]; it also holds other incoming axons, for example, from the pretectum and the contralateral tectum, as well as the dendrites and axons of tectal interneurons and projection neurons. The *Shh:GFP *transgenic line, in which all GCs express GFP [27,28], can be used to visualize the retinorecipient laminae. In a horizontal section, GC axons in *Shh:GFP *fish can be seen to innervate the stratum opticum (SO), three different sublaminae of the stratum fibrosum et griseum superficiale (SFGS), the stratum griseum centrale (SGC), and the border between the stratum album centrale (SAC) and stratum periventriculare (SPV) (Figure 2A). Four retinal input layers have also been described in adult goldfish [29]. As previously reported, *Pou4f3:mGFP*-labeled axons project to the SO and the two deeper sublaminae of the trilaminated SFGS (Figure 2D) [18].

Figure 2 shows the organization of Parv and PKC immunoreactivity in the tectum, with the *Pou4f3:mGFP *and *Shh:GFP *retinotectal inputs serving as a reference for the laminae of the tectal neuropil. PKC and Parv antibodies label two distinct populations of tectal neurons with distinct stratification patterns. PKC+ and Parv+ cell bodies have a rough spatial separation; PKC+ cells tend to be in the deeper portion of the SPV, and Parv+ cells lie close to the neuropil. Parv+ neurites are concentrated in five bands; three of these are co-localized with retinal input in the SO and SFGS, two lie in SGC and SAC. PKC+ neurites are concentrated in three laminae, in the SO, SFGS, and SGC. For both tectal markers, the precise location of each band varies between sections. In conclusion, cell type-specific labeling provides a convenient readout for synaptic lamination in the tectal neuropil.

### Disruptions of neuronal activity by dark rearing, amino-phosphono-butyrate, and *Botulinum *toxin B do not prevent IPL sublamination

**Figure 3:**
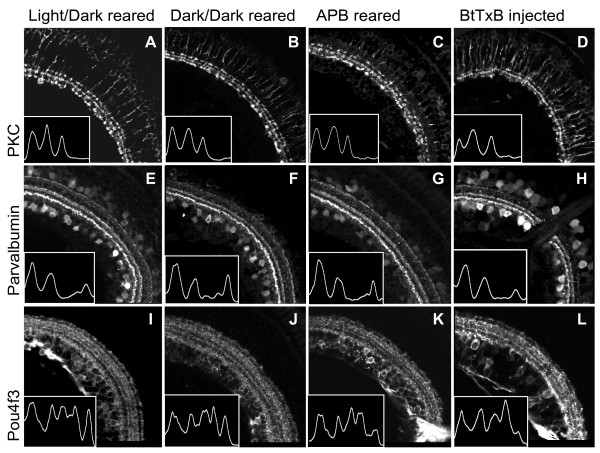
**Dark-reared, APB-treated, and BtTxB-injected larvae show proper IPL sublamination.(A-L) **Sections showing the IPL of 5 dpf larvae raised in a normal light:dark cycle **(A, E, I)**, constant darkness **(B, F, J)**, in the presence of 1 mM APB **(C, G, K)**, and treated with BtTxB **(D, H, L)**. The images in D, H, L are from the larva recorded in Figure 4D. Insets: traces of the fluorescent signal intensity across the width of the IPL (region shown). Peaks correspond to bands in the IPL. **(A-D) **PKC+ BC axon terminals are confined to three inner sublaminae in all larvae. **(E-H) **Parv+ neurites are in three bands in all larvae. The interruption of the IPL in H is the optic nerve. **(I-L)*** Pou4f3:mGFP+ *dendrites stratify in five bands in all larvae. Scale bar 50 μm.

Detection of light by photoreceptors shapes the patterns of synaptic activity in the IPL by 68 hours post-fertilization (hpf) [30], and is therefore poised to influence the stratification of IPL neurites. We began exploring the role of retinal activity in IPL development by testing the influence of visual experience. Zebrafish embryos were reared in complete darkness, beginning in early gastrulation. No qualitative alterations in the distribution of our markers were observed in dark reared larvae (Figure 3A, B, E, F, I, J; n = 2 larvae for PKC, 5 for Parv, and 8 for *Pou4f3:mGFP*). The stability of the pattern is demonstrated by the densitometric profiles of IPL staining patterns, shown as insets within their corresponding images in Figure 3. In these traces, pixel intensity is plotted versus distance from the proximal (inner) edge of the IPL, and IPL bands appear as peaks in pixel intensity.

**Figure 4:**
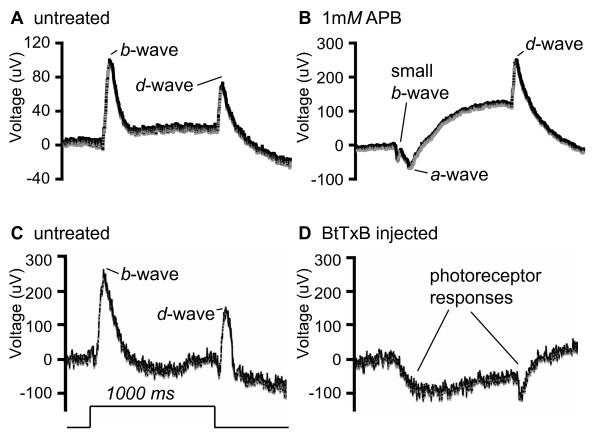
**Electroretinograms of APB- and BtTxB-treated larvae.** Representative averaged traces of larval responses to a 1s pulse of bright light. Note that absolute voltage is a function of electrode placement on the eye; these traces are best interpreted by comparing the amplitude of the downward *a*-wave to the upward *b*-wave, and the *on *to the *off *response. **(A, B) **Responses of untreated control and 1 mM APB treated larvae, in μV. **(C, D) **Responses of uninjected control and BtTxB injected larvae, in μV. Time course of light step stimulus is shown at the lower left.

In a second experiment, BC responses to the onset of light were inhibited with the mGluR6 agonist amino-phosphono-butyrate (APB). mGluR6 is a metabotropic glutamate receptor present on the dendritic terminals of *on-*type BCs; the receptor initiates a transduction cascade that hyperpolarizes the cell in the response to glutamate from photoreceptors, which release glutamate tonically in the dark [31]. As shown previously, APB blocks most of the retinal response to the onset of light in zebrafish, but none of the response to the offset of light [32,33]. We confirmed by electroretinogram (ERG) recordings that 1 mM APB blocked most of the *b*-wave, the bipolar-cell dependent *on *response, but did not attenuate the *d*-wave, the corresponding *off *response (Figure 4A, B) [34]. All of the 11 APB-treated animals recorded had responses similar to that shown in Figure 4B, wherein just a small residual *on *BC response is apparent within the larger downward response of the photoreceptors. The mean ratio of the amplitude of the *on *response to that of the *off *response in control animals was 1.9 (standard deviation 0.50 from 3 larvae). In APB-treated larvae, this mean ratio was reduced to 0.32 (standard deviation 0.18 from 11 larvae). Thus, APB treatment created a dramatic imbalance between *on *and *off *signals from BCs to ACs and GCs. Nevertheless, IPL sublamination was preserved in APB-reared larvae, as assessed by the labeling patterns of our three markers and the corresponding densitometric profiles (Figure 3C, G, K; n = 3 for PKC, 12 for Parv, and 10 for *Pou4f3:mGFP*).

Finally, we designed an experiment to block synaptic transmission across all cell types by injection of *Botulinum *toxin B (BtTxB) and asked if this treatment affected sublamination. This is an important test, because spontaneous activity of neurons, rather than experience-dependent activity, may be sufficient to assemble the correct connections [35]. BtTxB is a clostridial toxin that blocks synaptic vesicle fusion by cleaving the v-SNARE synaptobrevin [36]. As expected, injection at the single cell stage of BtTxB resulted in paralysis and sometimes malformation of the developing larva. In animals injected with sufficient BtTxB solution to cause permanent, total paralysis, the ERG *b*- and *d*-waves were severely depleted or abolished, whereas the *a-*wave, representing phototransduction currents, was still present (n = 4; Figure 4C, D). For our analysis, we selected only those animals that were completely paralyzed until 5 dpf, and included those that had confirmed BC response abrogation by ERG. In all of these animals, we could observe a qualitatively normal IPL sublamination pattern (Figure 3D, H, L; n = 3 for PKC, 11 for Parv, and 11 for *Pou4f3:mGFP*).

These experiments suggest that neither visual experience, nor a normal balance of *on *and *off *signals, nor chemical synaptic transmission are crucial to the formation of crisp IPL sublaminae. In the following sections, we report our analysis of possible quantitative changes to the IPL pattern resulting from these three treatments.

### Disruptions of activity lead to only subtle quantitative changes in IPL sublamination

**Figure 5:**
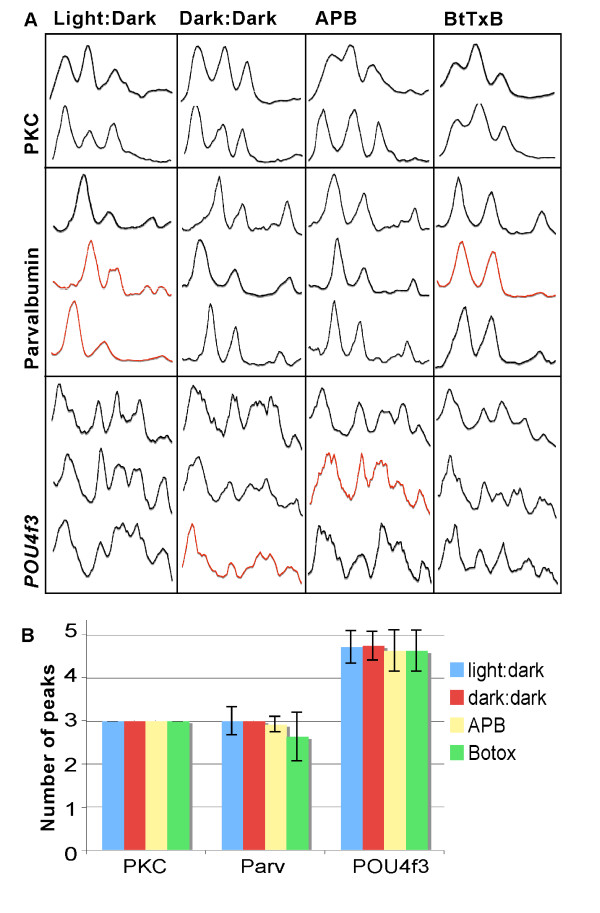
**IPL sublaminae are consistently preserved in activity-deprived larvae.(A) **A collection of Plot Profile traces representing IPL sublamination of different animals across all treatments (control, dark reared, APB, BtTxB; see Text). Each column corresponds to one rearing condition, shown at the top of the grid. **(B) **Quantification of the number of IPL sublaminae ('peaks' in a Plot Profile trace) observed for each of the three markers across the four rearing conditions. The number of PKC+ peaks was invariant; error bars for Parv+ and *Pou4f3:mGFP+ *peak numbers are 95% confidence intervals. Any significant difference between groups would generate non-overlapping error bars.

**Figure 6:**
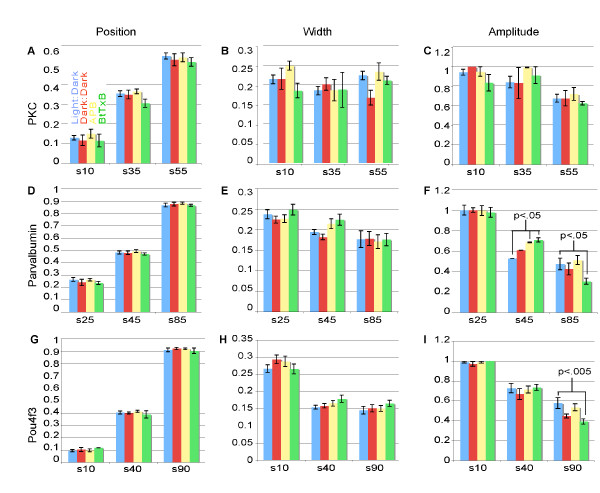
**Quantitative analysis of IPL sublamination in activity-deprived larvae.** Comparison of the mean locations, widths, and amplitudes of nine IPL bands across all treatments as in Figure 5. **(A, D, G) **Mean relative positions of PKC+ (top), Parv+ (middle), and *Pou4f3:mGFP+ *(bottom) sublaminae. The ordinate gives the distance from the inner edge of the IPL to the peak, divided by the total IPL width. **(B, E, H) **Mean widths of IPL sublaminae. The ordinate gives the width of the band (trough-to-trough on the densitometric trace) divided by the total IPL width, and therefore represents the fraction of the IPL covered by the given sublamina. **(C, F, I) **Mean relative amplitudes of the brightest pixels in each IPL band. The ordinate gives the amplitude (brightness) of the given peak divided by the maximum amplitude in the densitometric trace. All significant differences are labeled. Error bars show SEM.

The stereotypy of IPL sublamination allowed us to investigate if dark rearing, APB treatment, or BtTxB injection affected quantitative aspects of this pattern. For each marker and for each condition, we assessed the number of bands (peaks in the densitometric profiles), as well as the position, width, and brightness (amplitude) of each band (Figures 5 and 6). We observed in all experimental conditions the three bands formed by PKC+ BC axons, the three bands formed by Parv+ AC terminals, and the five dendritic strata from *Pou4f3:mGFP+ *GCs (Figure 5B). This suggests that the number and relative order of sublaminae is resilient to activity perturbations.

There also were no major, systematic differences between untreated, dark-reared, APB-treated, and BtxB-injected larvae in any of the parameters tested. The only possible exceptions were the amplitudes of the middle (s45) Parv band, which were increased in APB-reared and BtTxB-injected animals (*p *= 0.033 and *p *= 0.017, respectively; Figure 6F), and the amplitudes of the third (s85) Parv and the adjacent (s90) *Pou4f3:mGFP *bands, which were decreased in the BtTxB-injected animals (*p *= 0.010 and *p *< 0.005, respectively; Figure 6F, I). In fact, the s85 Parv band was below the detection threshold in 36% of BtTxB injected larvae compared to 11% of control, 0% of dark-reared, and 17% of APB-treated fish. Of the parameters tested, peak amplitude has the broadest range of interpretations, from level of gene expression, to number of arbor branches within the sublamina, to the targeting precision of neurites. It is unclear which of these variables determining labeling strength might be affected by APB or BtTxB.

Under all conditions, we noticed variability in the finer details of the IPL intensity profiles. In one final assessment of sublaminar targeting in the IPL, we therefore looked at the incidence of common aberrations in IPL organization across the four rearing conditions. The common aberrations are shown in red in the sample traces in Figure 5A. Double peaks in Parv band s85 were observed in 11% of untreated fish and 0% of all other fish; double peaks in *Pou4f3:mGFP *band s10 were observed in 9% of larvae from all groups except for dark-reared larvae, which had none. *Pou4f3:mGFP*+ bands s55 and s70 are often merged in the densitometric profiles; for this reason, position, width, and amplitude data were not collected for these two bands. However, the incidence of merged s55 and s70 bands (where the individual peaks were not distinct enough to reach the thresholds set to screen out noise) was not increased as a result of any of our treatments; 36% of untreated larvae showed this phenotype, compared with 17% of dark-reared larvae, and 36% again of both APB and BtTxB treated larvae.

In conclusion, the few changes we discovered in this study resulting from APB or BtTxB treatment all consist of mean amplitude changes of approximately 16–18%, and these mean amplitudes all fall within the range for untreated fish (for example, see the red Parv trace in the leftmost column of Figure 5A – an untreated fish with a very faint s85). On the other hand, the positions and widths of these bands, which more directly represent accurate neurite targeting, are preserved with remarkable precision. We therefore conclude that chemical synaptic transmission plays a minor, if any, role in IPL sublamination.

### Organization of retinorecipient layers in the tectum is resilient to activity disruption

**Figure 7:**
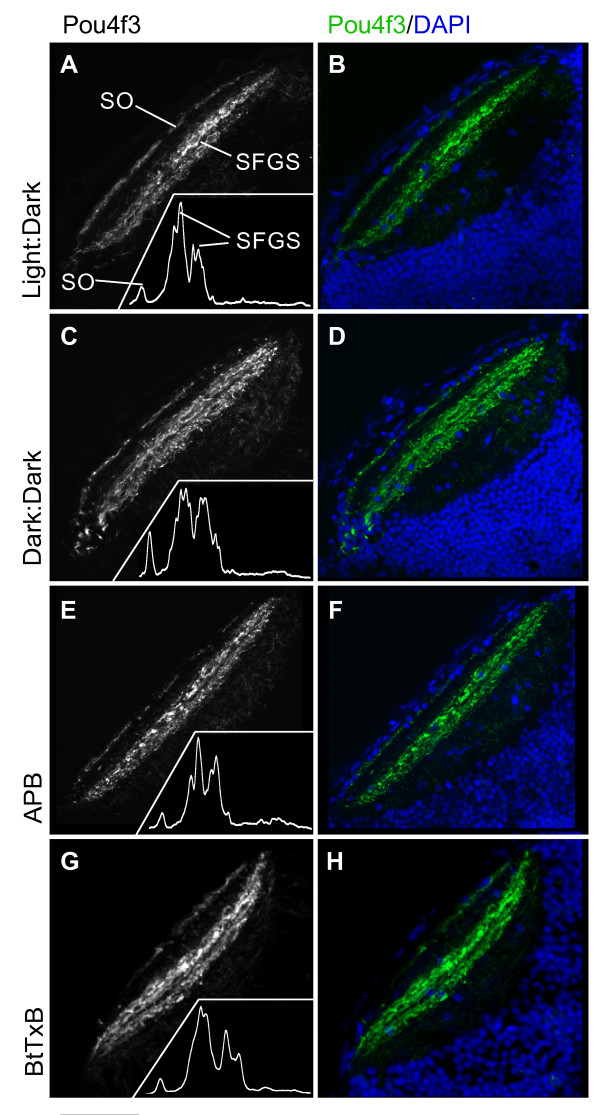
**Dark-reared, APB-treated, and BtTxB-injected larvae show proper GC axon targeting to tectal laminae.** Horizontal sections of 5 dpf larval tecta showing *Pou4f3:mGFP+ *GC axons innervating the optic tectum, imaged by confocal microscopy. **(A, C, E, G) ***Pou4f3:mGFP+ *axons innervate the SO and two sublaminae of the SFGS. Insets: densitometric traces across the tectal neuropil, from superficial to deeper layers. **(B, D, F, H) **Same images of *Pou4f3+ *axons (green), with DAPI labeling (blue) to show the cell body and neuropil regions of the tectum. Scale bar 50 μm.

Targeting to the tectal laminae SO and SFGS by *Pou4f3:mGFP*-labeled axons was investigated under each of the experimental conditions: in dark-reared fish, and following treatment with APB or BtTxB (Figure 7). There were no detectable differences between these treatments and controls, including the specific targeting of axons to the two closely apposed sublaminae in the SFGS, as demonstrated by the intensity profiles of GFP patterns in the tectum (insets in Figure 7). These data suggest that normal patterns of activity, including spontaneous waves of activity, are not required for the sorting of afferents into the retinorecipient layers of the tectum.

## Discussion

This study asked if the formation of stereotyped synaptic laminae in the retina and tectum was dependent on activity. We conducted several experiments in which the normal pattern of retinal activity was severely disrupted, but in each case the anatomy proved resilient to the experimental perturbation. First, removal of light inputs to the retina by dark rearing had no discernible effect on either the IPL or on the tectum. Because dark-reared zebrafish larvae perform a normal optokinetic response within a few minutes following their first exposure to light (our unpublished observations), sensory experience appears also dispensable for proper functional differentiation of synapses in the visual pathways. Second, APB-induced shifts in the relative activity of *off *and *on *BCs did not cause *on *BC terminals as a group to lose territory within the IPL; instead, no change was seen. Finally, blockade of chemical synaptic transmission with BtTxB left IPL and tectal lamination intact. (An important caveat here is that electrical synapses remain intact in BtTxB-treated animals and could potentially carry out a permissive role for activity.) The precision of this evidently hardwired process is remarkable, given that the layers examined are very fine – 1–3 μm in width in most cases. Indeed, the width of a single IPL sublamina corresponds to the diameter of a single GC dendrite and approaches the size of a presynaptic terminal.

We did detect small changes in the mean brightness of three bands under some conditions, but none brought the data from activity-deprived animals outside the range of values seen in untreated animals. From a wiring-specificity point of view, the parameters most relevant to neurite targeting are the number of bands and the width and position of each band; in these parameters, no significant differences were observed across all conditions in the expression of any of the three markers used.

None of our experiments excluded a role for activity *per se *in synaptic stratification. Other cell types and later developmental epochs remain to be tested; other manipulations could be carried out; and other readouts, including electrophysiological and behavioral ones, might be chosen to detect alterations of wiring precision. In particular, the distribution of synapses within laminae was not examined. Recent studies in the mouse have demonstrated a requirement for retinal activity in the number of varicosities on TH+ AC neurites in the IPL, and the columnar organization of certain GC axons in the superior colliculus [37,38]; our study would not detect changes in either of these aspects of wiring. Further, while the study of whole populations of neurons facilitates scoring neuropil lamination, the resulting data do not directly address the behavior of individual cells. Nevertheless, we have shown that patterns of chemical synaptic transmission are dispensable for laminar target selection by a large subset of zebrafish retinal neurites.

Our results suggest that retinal axons and dendrites use activity-independent cues – such as guidance and cell adhesion molecules – to sort into laminae. These cues could be cell surface molecules belonging to the immunoglobulin superfamily, such as Sidekick-1, Sidekick-2, Dscam, and Dscam-like [39,40], which have recently been shown to direct sublaminar specificity in the chick and mouse IPL. Adhesion molecules are also distributed in a lamina-specific pattern in the chick tectum, and molecularly defined GC subsets preferentially innervate distinct target laminae [41-43]. In an explant system of chick tectum where GCs had access to all laminae, the axons grew and arborized in a lamina-selective manner, even when the tectal explant was chemically fixed – suggesting that adhesion at the cell membrane is sufficient to guide GC axons [44]. Though no guidance molecule has yet been shown to direct tectal lamination, a zebrafish type IV collagen (Col4a5), which is an integral part of the basement membrane covering the tectum, is required for laminar targeting of GC axons. In the absence of Col4a5 function, repulsive factors normally anchored to the extracellular matrix are dispersed, allowing the deeper SFGS-projecting axons to trespass into the more superficial SO [45]. Forward genetic approaches in zebrafish may uncover additional guidance and adhesion molecules responsible for lamina-specific projections.

In the mouse, two phases of IPL development are known to be sensitive to retinal activity. First, prior to eye opening, waves of depolarization across the field of GCs play a role in converting many GC dendrite arbors from a diffuse to a refined morphology [46,47]. In a nicotinic acetylcholine receptor (*nAChRβ2*-/-) mutant mouse, which lacks this type of retinal wave, GC stratification is altered temporarily – dendrite arbors are delayed in their refinement [46]. After eye opening, the population of GC dendrite arbors, which tend to innervate the center of the IPL at eye opening, is rearranged in a light-dependent process, such that more arbors are stratified to the edges of the IPL weeks later [13]. Disrupted activity also leads to a delay in GC targeting in the cat retina; injection of APB into a kitten's eyes blocks the refinement of GCs to the mature, stratified state [2,10,11,48]. In all three cases, activity deprivation prevents GC dendrite maturation, such that the GCs at the end of the period have the morphology of younger GCs. Though GC development has been studied most thoroughly, dark rearing also delays the targeting of BCs and ACs [49,50]. In the mammalian brain, differences in the activity from the two eyes can also grossly alter the projection patterns of incoming axons in the lateral geniculate nucleus and visual cortex [51-54]. Thus, unlike what we have seen in zebrafish, activity clearly influences the formation of visual system synaptic laminae in mammals, although most of the activity-dependent changes seen in mammals can, to our knowledge, be attributed to delays in the normal wiring, rather than to abnormal wiring.

In contrast, consistent with our findings, a recent study using the *nAChRβ2*-/- mutant mouse revealed that the targeting of transient *off *alpha GCs to an SGC sublamina in the superior colliculus (the mammalian homologue of the tectum) was unaffected by the disruption of spontaneous retinal activity, even though the same axons required the normal pattern of activity to target specific intralaminar columns of approximately the same width [38]. Though the targeted tectal sublamina is a much larger structure than the IPL sublaminae presented here, the *nAChRβ2*-/- mouse result does underscore a shared conclusion: activity pattern-dependent and -independent mechanisms can direct neurite targeting with similar precision.

What could account for the species differences? A relevant difference between the mammalian models and the zebrafish is the pace of development. In the mouse, the fastest developing mammal in these studies, IPL assembly takes approximately 14 days (P0 to P14) [46,47], while in the zebrafish the equivalent process occurs in 2 days (2 to 4 dpf) [14,15,55]. Zebrafish GC stratification does not appear to be an accelerated version of its mammalian counterpart. In mammals, developing GCs extend diffuse dendritic arbors, which are subsequently refined to sublaminae [46,47,56-59]. In contrast, time-lapse confocal imaging revealed that the majority of zebrafish *Pou4f3:mGFP+ *GCs showed laminar specificity from the start of IPL innervation, though the initial pattern was often not the same as the final pattern [14]. One possible molecular interpretation for this distinction is that zebrafish GCs express a complement of adhesion molecules, guidance receptors, and their downstream effectors, enabling them to target sublaminae early on. The homologous genes may be expressed with a delay in the mammalian retina and may require induction through synaptic signaling. For instance, in mice and rats, neurons in the INL and GCL respond to light stimulation with activation of the constitutive transcription factor CREB and induction of the transcription factor genes *c-fos, c-jun, junB*, and *krox24 *[60]. It is conceivable that the target genes of these transcription factors include adhesion molecules or guidance receptors in addition to known regulators of synaptic strength. In this way, synaptic transmission of light signals could initiate a program that relies on hard-wiring cues to organize the IPL. The mechanisms of visual system lamination have clearly diverged between species, and the molecular details underlying this divergence remain to be discovered. Our study suggests that the established roles of synaptic transmission in mammalian visual pathway refinement are particular to that lineage; an organized IPL and tectal neuropil can be assembled by hardwiring mechanisms alone.

## Conclusion

In summary, our results show that normal synaptic signaling within the retina plays a minor role, if any, in laminar and sublaminar architecture in the zebrafish visual pathway. We observe that the zebrafish, which develops molecularly defined synaptic strata similar to those seen in mammals, can form a sublaminated IPL under the following experimental conditions: in the absence of light input; when the balance of *on *and *off *inputs is perturbed; and when chemical synaptic transmission is inhibited. Synaptic lamination in the larval zebrafish tectum shows similar resilience to these activity manipulations. Although we have not excluded effects of activity on cell types not sampled in our study or a permissive role of spontaneous activity transmitted through gap junctions, we think it is most likely that the placement of neurite endings within micron-width strata is accomplished largely by activity-independent mechanisms.

## Materials and methods

### Fish strains and transgenic lines

Adult zebrafish from the TL and WIK strains were maintained in our fish facility at the University of California, San Francisco. All procedures adhered to the rules of animal use set by the National Institutes of Health and UCSF. Transgenic lines, generated and maintained in TL, were *Pou4f3:mGFP *[*Tg*(*Pou4f3:gap43-GFP*)^*s*273*t*^] [18], and *Pax6:mGFP *[*Tg*(*Pax6-DF4:gap43-GFP*)^*s*220*t*^] [16].

### Sectioning

Larvae aged 5 dpf were fixed in 4% paraformaldehyde (PFA; w/v, pH 7.4) overnight at 4°C, infiltrated in 30% sucrose (w/v) in phosphate-buffered saline (PBS) overnight at 4°C, and embedded in molds containing OCT freezing medium (Sakura Finetech USA, Inc., Torrance, CA, USA). In all cases, treated larvae were embedded beside untreated siblings in reverse orientation for comparison. Blocks were then frozen at -20°C. Embedded larvae were sectioned horizontally on a Jung Frigocut 2800N cryostat (Leica Instruments, Nussloch, Germany). The 12 μm sections were collected on Superfrost Plus slides (Fisher Scientific, Pittsburgh, PA, USA), air dried for 30 minutes to overnight, and re-hydrated in PBS.

### Immunohistochemistry

Sections were incubated with blocking reagent containing 3% (v/v) normal goat or donkey serum (Jackson ImmunoResearch Laboratories, Westgrove, PA, USA) and 0.3% Triton X-100 (v/v; Fisher Scientific) in PBS (pH 7.4) for 30 minutes at room temperature. Primary antibodies were diluted in blocking solution and pipetted onto sections; slides were left overnight in primary antibody at 4°C in a humidified chamber. The following day, sections were washed three times in PBS and then incubated for 2 h in a solution of Alexa fluorophore-conjugated secondary antibody (10 μg/ml in blocking solution; Invitrogen Molecular Probes, Eugene, OR, USA). Finally, sections were washed in PBS as above, stained with DAPI nuclear marker (Sigma, St Louis, MO, USA), and mounted in Fluoromount G (Southern Biotechnology Associates, Inc., Birmingham, AL, USA) under microscope coverslips (Fisher Scientific). Slides were air-dried in the dark from 4 h to overnight.

### Primary antibodies used

Primary antibodies used were: goat anti-ChAT (1:50; Chemicon, Temecula, CA, USA); mouse anti-Parv (1:1200; Chemicon); rabbit anti-PKC β1 (1:800; Santa Cruz Biotechnology, Santa Cruz, CA, USA); mouse anti-TH (1:400; Chemicon); rabbit anti-GFP antibody (1:4000; Invitrogen Molecular Probes); and zrf3 (1:250; Oregon Monoclonal Bank, Eugene, OR, USA).

### Imaging sections

Confocal images were captured using a Zeiss LSM 5 Pascal microscope and software. Confocal stacks were further processed using ImageJ software . In some cases, z-projections of a few slices were made; in others, single representative slices were selected. In all figures, comparisons are made between images that were processed equivalently – slices compared to slices, and projections compared to projections of a similar number of slices. Fluorescence images were adjusted in Adobe Photoshop using the brightness/contrast, levels, and curves functions in order to best represent the pattern of neurite lamination.

### Dark rearing of larvae

Progeny from in-crosses of *Pou4f3:mGFP *transgenic adults were raised from 12 hpf in dishes wrapped in aluminum foil. At 5 dpf, the foil was removed in a dimly lit room and larvae were immediately fixed in 4% PFA.

### APB rearing

Progeny from in-crosses of *Pou4f3:mGFP *transgenic adults were dechorionated at 30 hpf and moved to E3 embryo medium containing 1 mM D, L-APB (Sigma) (5 mM NaCl, 0.17 mM KCl, 0.33 mM CaCl_2_, 0.33 mM MgSO_4 _supplemented with 1:10^7 ^w/v methylene blue). The medium was changed daily to maintain the efficacy of the drug. (The same ERG result was obtained in an experiment where we did not replace the solution.) At 5 dpf, larvae were assessed by ERG recording or fixed in 4% PFA for histological analysis.

### *Botulinum *toxin B injection

Progeny from in-crosses of *Pou4f3:mGFP *transgenic adults were injected at the one-cell stage with an approximately 5 nl bolus of 1.0 ng/nl BtTxB (EMD Biosciences, Darmstadt, Germany). At 5 dpf, immobilized larvae were tested extensively for startle responses and sectioned; a subset were tested for retinal neural activity by ERG recording prior to sectioning.

### Quantitative analysis of the IPL

All image analysis was performed blind to treatment category of the imaged section. For each larva, the most central section of the retina for each antibody stain was selected for analysis. To make the densitometric profile plots, a rectangular region of interest was drawn across a short, relatively straight stretch of the IPL or neuropil with pronounced sublaminae, using the DAPI stain to see the IPL edges. ImageJ's Plot Profile function was applied to the rectangle, to calculate an average fluorescence intensity trace across the IPL or neuropil width. Labeled laminae appear as peaks in the traces. The traces were imported as numeric data into Excel. All data were normalized to the maxima, such that position represents the distance of the peak maximum from the proximal edge of the IPL divided by the total IPL width, and amplitude is the pixel intensity relative to the maximum pixel intensity in the selected portion of the image. Width refers to the fraction of the IPL width covered by the peak (from trough to trough). To count peak numbers, empirically determined criteria – a threshold width, height, and rise – were applied uniformly to all data to distinguish noise in the traces from real IPL bands. All bands were analyzed except for the third and fourth bands of *Pou4f3:mGFP+ *dendrites (s55 and s70), which tended to be insufficiently separated. Data for each parameter (location, width, and amplitude) of each IPL band were compared across the four rearing conditions (normal light:dark cycle, constant darkness, APB, BtTxB) using a one-way ANOVA. Where significant group effects were found, pairwise *t*-tests were used to find which conditions differed significantly from the untreated (light:dark reared) controls.

### Electroretinogram recording and analysis

Untreated fish were tested at the beginning and end of each session to confirm the in-session consistency of the recording equipment; we found that these first and last control recordings were always equivalent. Anaesthetized larvae were mounted on their sides on a foam platform in the recording chamber, and the recording electrode was placed against the lens. Recordings were made under scotopic conditions (normal indoor lighting). Larvae were presented with a series of eight 1s duration 500 μW light steps, with 10s intervals. These step stimuli elicit an *on *response followed by an *off *response at light offset. For each larva, the eight traces were averaged to generate the curves shown in Figure 4. For quantification of the effect of APB, the amplitudes of the *on *and *off *responses were measured relative to the trough immediately preceding each peak. This was important because the APB-treated *on *responses were small positive responses within negative *a-*waves. For each animal, the ratio of *on *to *off *amplitude was calculated for a single averaged trace.

## Abbreviations

AC: amacrine cell; APB: amino-phosphono-butyrate; BC: bipolar cell; BtTxB: *Botulinum *toxin B; ChAT: choline acetyltransferase; dpf: days post-fertilization; ERG: electroretinogram; GC: ganglion cell; GCL: ganglion cell layer; GFP: green fluorescent protein; hpf: hours post-fertilization; INL: inner nuclear layer; IPL: inner plexiform layer; ONL: outer nuclear layer; Parv: parvalbumin; PBS: phosphate-buffered saline; PFA: paraformaldehyde; PKC: protein kinase C; SAC: stratum album centrale; SFGS: stratum fibrosum et griseum superficiale; SGC: stratum griseum centrale; SO: stratum opticum; SPV: stratum periventriculare; TH: tyrosine hydroxylase.

## Competing interests

The authors declare that they have no competing interests.

## Authors' contributions

LMN carried out experiments, histology, imaging, data analysis, and co-drafted the manuscript. MRT developed and recorded ERGs. HB conceived of the study, and participated in its design and coordination and co-drafted the manuscript. All authors read and approved the final manuscript.

## Supplementary Material

Additional file 1 Tyrosine hydroxylase-positive neurites innervate the edges of the IPL. Sectioned 5 dpf retina immunostained to tyrosine hydroxylase (TH), imaged by wide-field fluorescence microscopy, shows small processes at the inner plexiform layer edges (arrows). The photoreceptor autofluorescence is particularly high in this image; the photoreceptors are not TH immunopositive. Scale bar 50 μm. Click here for file
