# Supplementary figures and images for: Hardwiring of fine synaptic layers in the zebrafish visual pathway
Source: Neural Dev. 2008 Dec 16;3:36. doi: 10.1186/1749-8104-3-36 (PMC2647910; doi:10.1186/1749-8104-3-36)

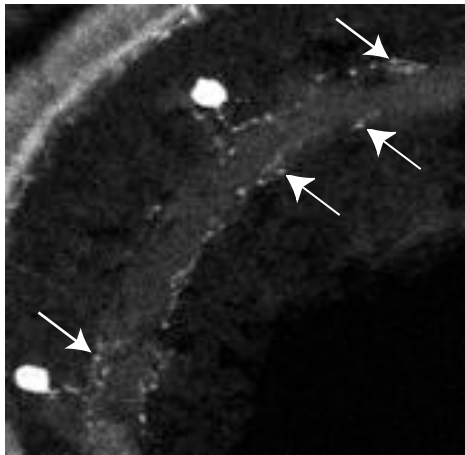

Supplement: Additional file 1 — Tyrosine hydroxylase-positive neurites innervate the edges of the IPL. Sectioned 5 dpf retina immunostained to tyrosine hydroxylase (TH), imaged by wide-field fluorescence microscopy, shows small processes at the inner plexiform layer edges (arrows). The photoreceptor autofluorescence is particularly high in this image; the photoreceptors are not TH immunopositive. Scale bar 50 μm. [file 1749-8104-3-36-S1.pdf]
